# Supplementary material for: Improvement in sensitivity of radiochromic 3D dosimeter based on rigid polyurethane resin by incorporating tartrazine
Source: PLoS One. 2020 Mar 16;15(3):e0230410. doi: 10.1371/journal.pone.0230410 (PMC7075553; doi:10.1371/journal.pone.0230410)
Supplement: Attachment — Submitted filename: Point to point response_submitted.docx [file pone.0230410.s001.docx]

**Reviewer #1**:

Introduction

1. Line 61. In the clinical practice, 3D dosimetry is mainly performed with dosimeters other than chemical ones, for example by moving ionization chambers or silicone diodes or TLDs etc. in different positions of e.g. a water phantom (take a look at the following publication:<https://www.ncbi.nlm.nih.gov/pmc/articles/PMC5368627/>). The statement al line 61 should be rearranged.

- We appreciate the reviewer’s comments. We have added the description performing 3D dosimetry using ionization chambers or silicone diodes or TLDs.

2. Line 63. This statement is not clear since optical absorption, scattering, X-ray absorption, NMR are not properties of dosimeters, but physical phenomena.

- We appreciate the reviewer’s point. It was corrected in the revised manuscript. We have modified “Physical response” to “physical phenomena”.

3. Line 78. “This change in optical density (OD) is linear with respect to the absorbed dose up to 100 Gy”. This statement is uncomplete since it is important to specify the full range (linear in the range…).

- We appreciate the reviewer’s point. It was corrected in the revised manuscript.

4. Lines 93-99. This paragraph is not well linked to the rest of the introduction. Additional sentence(s) aiming at introducing the reason for discussing EBT and EBT2 films are needed. Furthermore, the sentences of lines 96-98 must be better explained; the conclusions are faint and should be supported by more well-founded arguments.

- As the reviewer pointed out, it was corrected in the revised manuscript. We have added additional descriptions to link sentences explaining the reason for discussing EBT and EBT2 films.

5. Lines 100-102. Also this paragraph needs a better introduction. For example, it is not said why the authors choose to add just the tartrazine as a yellow dye. What is its chemical structure, are there similarities with the dye of EBT films?

- As the reviewer recommended, it was modified in the revised manuscript. We appreciate the reviewer’s comments. We have added the descriptions about tartrazine among yellow dyes. The yellow dyes used for Gafchormic^TM^ radiochromic film could be tartrazine, eosin, quinoline yellow, metanil yellow. Particularly, tartrazine is useful dye among yellow dye according to manufacturer. Tartrazine is classified by azo compounds which are used as free radical initiators. An azo compound has an azo group (two nitrogen atoms joined by a double bond, R-N=N-R') which is decomposed by heat and/or light, and forms carbon radical. The formed carbon radical is excellent in reactivity, and progresses polymerization and halogenation reaction of different types of vinyl monomers. The sensitivity of PRESAGE dosimeter was related to concentration of free radical initiators. Therefore, we thought that the added tartrazine could be act as an initiator. However, the effect of the tartrazine as free radical initiators on the dosimetric characteristics of PRESAGE dosimeter has not been investigated.

Figure. Chemical structure of tartrazine

Materials and Methods

6. Line 106. “Radiochromic plastic gels were fabricated…”. Did the authors fabricated commercial PRESAGE dosimeters with the addition of tartrazine or they developed new dosimeters?

- We appreciate the reviewer’s comments. It was modified in the revised manuscript. We fabricated the dosimeters with compositions of the commercial PRESAGE dosimeter.

7. Line 157. Was the uniformity of the radiation field measured/calculated? The uniformity is generally expressed with a percentage.

- We appreciate the reviewer’s comments, it was corrected in the revised manuscript. We calculated the uniformity of the radiation filed with Eclipse™ system. The uniformity was ±1.0% of delivering dose.

8. Line 161. Monitor Unit is not physical quantity. Gy/min and its multiples/submultiples are the correct units.

- As the reviewer pointed out, it was corrected in the revised manuscript.

9. Lines 172-173. From this sentence, I understand that PRESAGE dosimeters change the absorbance for exposure to VIS light. Since this aspect is crucial for the use of PRESAGE dosimeters in the practice, a comment is needed.

- We appreciate the reviewer’s comments and agree with the reviewer’s comments. We also maintained a dark environment when manufacturing and storing the dosimeters. The reference for storage and handling was added.

10. Line 179. Radiochromic films are sensitive to UV light, namely they show an increasing darkness with the increase of the exposure to UV light. I expect a same behaviour for PRESAGE dosimeters. Since the Xe flash lamp emits in the range 200-830 nm, could this be an issue for this work? Can the authors comment on this aspect?

- We appreciate the reviewer’s comments. The reading method using the spectrometer of the presage dosimeter is obtaining absorbance by transmitting light similarly to a film scanner. As the reviewer mentioned, the color changes when exposed to spectrometer light, but the dosimeters once measured with spectrometer was not reused.

11. Lines 199-207. It is not clear which is the actual output quantity of the spectrometer (Absorbance, Net absorbance?); how the other quantities are evaluated and how the spatial information (delta Absorbance/Gy*cm) in the dose sensitivity is evaluated. Optical density and absorbance are usually used to describe the same quantity, while in this manuscript they seem to indicate different quantities (“The dose response curve was obtained by plotting optical density and maximum absorbance as a linear function of absorbed dose.” and “using optical absorbance and optical density measurements” in the conclusions). These points are crucial for the understanding of all the manuscript and need to be rewritten.

- We appreciate the reviewer’s comments. Actual output quantity of the spectrometer and optical-CT scanner was absorbance as known the optical density (OD). However, we distinguished "absorbance" measured with the spectrometer and "optical density" measured with the optical CT scanner. The change in absorbance is delta absorbance (Δabsorbance) which was incorrectly written as net absorbance. We modified net absorbance with Δabsorbance. The change in absorbance (Δabsorbance) values at λ_max_ were obtained by subtracting the absorbance value at λ_max_ for the un-irradiated cuvette from that of the irradiated cuvettes. From the optical absorption with spectrometer, the dose sensitivity (Δabsorbance/(Gy·cm)) was defined as the slope of the dose response curve, which was the maximum Δabsorbance at λ_max_ with absorbed doses. The change in optical density (ΔOD) was obtained by subtracting the OD_0_ for the un-irradiated cuvette from that of the un-irradiated cuvettes. For 3D reconstructed images obtained from optical CT scanner and software, the dose sensitivity (ΔOD/(Gy·cm)) was defined as the slope of the dose response curve, which was the ΔOD with the absorbed doses. The dose response curve was obtained by plotting the ΔOD with optical CT scanner and maximum Δabsorbance value at λ_max_ with spectrometer as a linear function of absorbed dose.

Results

12. Lines 223-227. This presentation of results (Zeff for 2 formulations and presence of some elements in the compound) is meaningless. It would be interesting to see the results (tables, plots etc.) of XRF measurements for all (or at least for some) formulations. Moreover, it is said the chemical composition includes C, H, O, N and Br. However, no results are given for the concentration of these elements. This is a useful information for possible further studies on these dosimeters.

- As the reviewer recommended, it was added in the revised manuscript. The results of XRF measurements for all formulations was added.

13. Line 231. “Very stable” is meaningless. Can the authors quantify how the spectra are stable after irradiation?

- We appreciate the reviewer’s point. We referred to paper in revised manuscript. With regard to post irradiation of PRESAGE dosimeter, Guo et al. reported that can be stable (∼2%) during 2 hours to 2 days post irradiation. Based on results, we performed reading using spectrometer and optical CT after 2 hours post-irradiation. Since the sensitivity was relatively evaluated for the same condition, reading was also performed at the point where there was no significant change.

14. Line 232. How the results are consistent with OD measurements? Are OD measurements those performed with CT scanning (see comments at line 199-207)?

- We appreciate the reviewer’s point. When we measured post irradiation of fabricated dosimeters with optical CT scanner, we also obtained the result that there was no significant OD change after 2 hour post irradiation.

15. Lines 232-242. Scientific results should be presented in plots or tables which make clear the text.

- As the reviewer pointed out, the Fig 3 was modified to explain the results in revised manuscript.

16. Table 2. Express correctly the significant figures of the quantity “sensitivity at lambda max” in table 2.

- As the reviewer pointed out, it was corrected in the revised manuscript.

17. Lines 299-301. This sentence is not clear.

- We appreciate the reviewer’s comments. The ratio of absolute OD values between the dosimeters using the same polyurethane was also the highest at low doses and the tendency was shown to decrease as the dose increased. By incorporating tartrazine, the absolute OD of all fabricated dosimeters was increased. The ratio between CC200 and CC200T was the largest among other formulations. This result is consistent with the most prominent tartrazine effect at CC200T having the highest dose sensitivity.

18. Discussion

Also in this case, the reading is very hard. It is not clear which is the point. A synthesis will help.

- We appreciate the reviewer’s comments. It was corrected in the revised manuscript. We have shortened the discussion on EBT films to give a rationale for using a yellow dye with a PRESAGE type dosimeter.

19. Conclusion

What is the sensitivity enhancement of the most performing fabricated dosimeter compared to the commercial PRESAGE dosimeter? It would be useful to report this information in the conclusion.

- As the reviewer recommended, it was corrected in the revised manuscript. The sensitivity was enhanced in the dosimeter with tartrazine compared with the commercial PRESAGE dosimeter, particularly CC200T.

**Reviewer #2:**

1. Could you explain the physical mechanism for the increase of absorption by adding tartrazine? Yellow dye was added to EBT2 to make it less sensitive to room light as you mention in the introduction.

- We appreciate the reviewer’s comments. Tartrazine is classified by azo compounds which are used as free radical initiators. An azo compound has an azo group (two nitrogen atoms joined by a double bond, R-N=N-R') which is decomposed by heat and/or light, and forms carbon radical. The formed carbon radical is excellent in reactivity, and progresses polymerization and halogenation reaction of different types of vinyl monomers. The sensitivity of PRESAGE dosimeter was regard to concentration of free radical initiators. Therefore, we thought that the added tartrazine could be act as an initiator.

2. You need to mention why one needs a 3D dosimeter with higher radiation sensitivity in the Introduction.

- As the reviewer recommended, it was corrected in the revised manuscript. We have added the necessity of dosimeter with a high dose sensitivity that can accurately measure dose distribution with very steep dose gradients.

3. The effective Z of the dosimeter material was determined to be 10.5, which is much higher than that of water, which is about 7.4 at the X-ray energy used for imaging such as CT. Are you concerned about the high Z-effective as a dosimeter used in radiation oncology?

- We appreciate the reviewer’s comments. Because the dosimeter has a relatively high effective atomic number compared to that of water or human tissues, it's not tissue equivalence. For relatively low energy X-Ray beams (30 keV-25 MeV), the predominant interaction in materials of low effective atomic number is the Compton Effect. As for a high effective atomic number material, there exists a higher probability of secondary electron generation through the photoelectric effect. Hence, during the measurement of depth-dose, a different depth-dose curve from it measured with the ionization chamber in water is obtained. The dosimeter was required to perform calibration to utilize 3D dose QA for IMRT or VMAT and for accurate measurement of the absolute dose.

4. XRF provides us atomic compositions. How did you calculate Z-eff? What formula did you use? What photon energy did you use to calculate it?

- We appreciate the reviewer’s comments. We used the method by Mayneord in a discussion of the Röntgen.

$$Z_{eff}=\sqrt[m]{\sum f_{i}Z_{i}^{m}}$$

The relative electron fraction of the *i* th element Z_i_ is given by f_i_. Mayneord used a value of 2.94 for the exponent *m*. We analyzed the dosimeter with XRF by using in the energy range from 0.1 -60.0 keV.

5. The discussion on EBT films in lines 337-365 should be shortened. Only a reason it is here is to give a rationale for using a yellow dye with a PRESAGE type dosimeter.

- We appreciate the reviewer’s comments. We have revised and shortened the discussion on EBT films to provide evidence for using a yellow dye.
